# Primary care online training on multifactorial breast cancer risk: pre–post evaluation study

**DOI:** 10.3399/BJGPO.2024.0305

**Published:** 2025-09-10

**Authors:** Francisca Stutzin Donoso, Juliet A Usher-Smith, Lorenzo Ficorella, Antonis C Antoniou, Jon Emery, Marc Tischkowitz, Tim Carver, Douglas F Easton, Fiona M Walter, Stephanie Archer

**Affiliations:** 1 Primary Care Unit, Department of Public Health and Primary Care, University of Cambridge, Cambridge, UK; 2 Centre for Cancer Genetic Epidemiology, Department of Public Health and Primary Care, University of Cambridge, Cambridge, UK; 3 Centre for Cancer Research and Department of General Practice and Primary Care, University of Melbourne, Melbourne, Australia; 4 Department of Genomic Medicine, National Institute for Health Research Cambridge Biomedical Research Centre, University of Cambridge, Cambridge, UK; 5 Centre for Cancer Genetic Epidemiology, Department of Oncology, University of Cambridge, Cambridge, UK; 6 Wolfson Institute of Population Health, Barts and the London School of Medicine and Dentistry, Queen Mary University of London, London, UK; 7 Department of Psychology, University of Cambridge, Cambridge, UK

**Keywords:** primary health care, e-learning, breast cancer, breast neoplasms, risk assessment

## Abstract

**Background:**

It is estimated that more than 250 000 women in the UK are at increased risk of breast cancer, but only a small fraction are identified. Digital tools, such as CanRisk, enable multifactorial breast cancer risk assessment. Implementation of such tools within primary care would allow primary care professionals (PCPs) to reassure women at population-level risk and identify those at increased risk who will benefit most from targeted prevention or early detection. Previous studies suggest that PCPs will require educational resources to support the delivery of multifactorial breast cancer risk assessments.

**Aim:**

To develop and evaluate a new ‘Multifactorial breast cancer risk assessment in primary care’ online training for UK PCPs.

**Design & setting:**

A mixed-methods pre–post evaluation study was undertaken. Thirty-five PCPs from across the UK participated in the evaluation and data collection was completed online between May and July 2024.

**Method:**

The online training was developed following a scoping review of the literature. The Kirkpatrick model of training evaluation was used as a framework and participants were given pre-training and post-training evaluation questionnaires. Statistical analysis for the evaluation focused on the primary outcome of objective knowledge and mean changes were analysed with a paired sample *t*-test. Qualitative feedback was analysed using content analysis.

**Results:**

Objective knowledge showed a significant mean increase (0.771, 95% confidence interval [CI] = 0.187 to 1.355, *P* = 0.011). Subjective knowledge and confidence scores also showed significant mean increases (6.828, 95% CI = 5.150 to 8.506, *P*<0.001; 4.085, 95% CI = 2.764 to 5.406, *P*<0.001, respectively). Results on satisfaction, engagement, and relevance of the training were positive.

**Conclusion:**

The ‘Multifactorial breast cancer risk assessment in primary care’ online training significantly increases PCPs’ knowledge and confidence to conduct multifactorial breast cancer risk assessments, and it was well received by PCPs.

## How this fits in

It is estimated that more than 250 000 women in the UK are at moderate or high-risk of breast cancer and eligible for risk-reducing medication or enhanced screening, but only a small fraction are identified. Incorporating multifactorial breast cancer risk assessment within primary care using tools, such as CanRisk, would allow primary care professionals (PCPs) to reassure women at population-level risk and identify those at increased risk who would benefit most from available interventions. A known limitation to implementing multifactorial breast cancer risk assessment is PCPs’ knowledge and confidence regarding genomics and cancer risk prediction methods and outcomes. The ‘Multifactorial breast cancer risk assessment in primary care’ online training introduced in this article significantly increased PCPs’ knowledge and confidence to conduct multifactorial breast cancer risk assessments, and it was well received by PCPs.

## Introduction

It is estimated that >250 000 women in the UK are at moderate or high-risk of breast cancer and are eligible for risk-reducing interventions (for example, risk-reducing medication, enhanced screening, or surgery).^1,2^ Until recently, identifying those at increased risk of developing breast cancer in the absence of a relative with a known pathogenic variant in a breast cancer predisposition gene (for example, BRCA1) relied largely on women opportunistically contacting their GP. This meant that only a small fraction of women at increased risk were being identified.^1,3^ Changes to the National Institute for Health and Care Excellence (NICE) guidelines (CG164) in 2022, which removed the recommendation against proactive assessment of breast cancer risk, may result in an increase in the number of risk assessments being conducted in primary care.^2^

There are several risk models and tools that facilitate the assessment of a woman’s risk of developing breast cancer.^4^ The CanRisk tool is a web interface for the validated Breast and Ovarian Analysis of Disease Incidence and Carrier Estimation Algorithm (BOADICEA), which enables healthcare professionals to conduct risk assessments.^5–9^ CanRisk provides an estimate of a woman’s future risk of breast cancer based on multiple genetic and non-genetic risk factors, including family history, lifestyle or hormonal and polygenic scores. CanRisk is currently used in many NHS specialist genetics clinics but is less widely used in primary care.

A known limitation to implementing multifactorial breast cancer risk assessment is primary care professionals’ (PCPs’) knowledge and confidence regarding basic genetics and genetic testing, as well as cancer risk prediction methods and outcomes.^10–15^ Research shows that PCPs have varied levels of knowledge about statistics and risk, have problems understanding complex risk outputs,^16–18^ and in some cases, find it difficult to communicate risk.^18–22^ When combined, these factors reduce the perceived value of risk assessment tools for PCPs and limits their motivation to use them.^16^ Encouragingly, recent research has shown that PCPs are interested in learning more about the target population for genetic testing, tests available, counselling, and risk communication strategies.^23,24^

Adopting new ways of working can also be challenging and requires healthcare professionals to learn how to navigate new systems and implement innovations.^25^ Motivation to implement a healthcare innovation needs it to be viewed as meaningful or having clinical utility.^6,26^ Knowledge, skills, and motivation are therefore closely intertwined, and any training aimed at improving knowledge and confidence also needs to include a clear and strong message regarding the potential utility of conducting breast cancer risk assessment using CanRisk in primary care.

Training to increase knowledge and confidence regarding genetics in general has proven useful in the past,^27,28^ and a systematic review of the effectiveness of educational intervention types to improve genomic competency in non-geneticist clinicians showed statistically significant effects especially to increase confidence.^29^ Consistently, training on shared decision making and risk communication in clinical settings has proven effective in increasing confidence and some aspects of objective knowledge.^22^

E-learning or online training methods to address the needs of clinicians on matters relevant to breast cancer risk assessments have been described as acceptable, practical,^30^ and effective.^22,31^ This study aimed to develop and evaluate an online training programme to support UK PCPs in the delivery of multifactorial breast cancer risk assessment in primary care. Specific objectives were to:

1. develop an evidence-based online training alongside stakeholder input;
2. evaluate PCPs’ satisfaction, engagement, and relevance of the online training; and
3. evaluate PCPs’ subjective and objective knowledge and confidence around multifactorial breast cancer risk assessment before and after they completed the online training.

## Method

### Development of the training

The content and presentation of the ‘Multifactorial breast cancer risk assessment in primary care’ online training was developed following a scoping review of the literature and stakeholders’ feedback.

### Evaluation study design

The evaluation of the training was a mixed-methods pre–post study.

### Evaluation framework

We used the Kirkpatrick model of training evaluation as a framework.^32^ The Kirkpatrick model is a well-established framework to evaluate training in different learning environments^33^ and has been recently revised in view of the current easy access to information and learning through the web and e-learning.^32^ The model has the following four levels: reaction (1), learning (2), behaviour (3), and results (4).

In line with the evaluation strategy of national education and training programmes (that is, NHS England), this study was focused on assessing levels 1 and 2. Currently unavailable real-world long-term scenarios are required to assess levels 3 and 4.

The sub-areas covered in level 1 were evaluated in study objective 2. Level 1 (‘reaction’) is defined as the degree to which participants find the training favourable, engaging, and relevant to their job. Thus, this level of the evaluation focuses on capturing participants’ views on ‘satisfaction’, ‘engagement’, and perceived ‘relevance’ of the training.^32^

The sub-areas of ‘knowledge’ and ‘confidence’ in level 2 (‘learning’) were evaluated in study objective 3. ‘Knowledge’ is defined as *‘the degree to which participants know certain information’*.^32^ ‘Confidence’ is defined as the degree to which participants think they are able to complete a particular task or will be able to do what they learnt on the training in their job.^32^

### Measures

The pre-training evaluation questionnaire (Supplementary Material S1) included participants’ demographic information, and questions focused on baseline objective and subjective knowledge of, and confidence in, conducting multifactorial breast cancer risk assessment. We distinguished between ‘objective’ and ‘subjective’ knowledge, namely knowledge that is measured through factual tests and self-reported expertise, respectively.^34^ The post-training evaluation questionnaire (Supplementary Material S2) included the same questions plus questions on participants’ reactions to the online training (assessing Kirkpatrick level 1) and two open-text questions asking for participants’ general views on the training and opportunities for improvement. Questions assessing objective knowledge and reaction to the training, respectively, were designed by the team of experts from the CanRisk team who took part in the development of the training (Table 1). Questions on subjective knowledge, confidence, and reaction to the training were validated questions from the Kirkpatrick framework.^32^

**Table 1.** ‘Multifactorial breast cancer risk assessment in primary care’ online training

Development of the training	Training objectives	Description of the training
– Scoping review was led by FSD and the initial content plan was supported by subject matter experts from the CanRisk team (ACA, MT, JAUS, FMW, JE, SA). – Content plan was reviewed by and discussed with members of our general practice expert advisory panel. – The panel is composed of seven GPs and three practice nurses that support the development of the CanRisk Cancer Research UK programme grant (PPRPGM-Nov20\100002).	To understand the basic components of the CanRisk multifactorial cancer risk prediction model. To recognise how genetic testing and multifactorial risk prediction for breast cancer works in practice. To communicate the results of multifactorial breast cancer risk prediction in primary care. To identify the outcomes and management options for each risk group identified through multifactorial breast cancer risk prediction.	– Four educational videos each covering one of the learning objectives featuring experts from the CanRisk team: ACA in risk prediction; MT in cancer genetics; JE in risk communication in primary care; and JAUS in cancer risk management in primary care. – There is also a brief introduction video with an overview of training (SA) and a brief ‘main takeaway’ video summing up the content of the training and why it matters (FSD). – The videos are 40 minutes long in total. – The training also includes a selection of further relevant online educational resources from elearning for health care (e-lfh.org.uk).

Abbreviations refer to authors’ initials.

### Procedure

A recruitment company (Sermo) completed the recruitment of 35 participants in total, 18 GP and 17 practice nurses (PNs). A balanced number of GPs and PNs practising in the UK were purposively sampled to include a broad range of years of experience working in primary care and, if possible, varying levels of familiarity and confidence using digital risk assessments tools (Table 2). Sermo had a set incentive rate at the time of £90 for participants who completed the entire evaluation.

The participants undertook the following three steps.

**Table 2.** Characteristics of participants and GP practices

	*N* = 35	%
**Participants**		
**Profession**		
General Practitioner	18	51.42
Practice Nurse	17	48.57
Gender		
Female	22	62.85
Male	13	37.14
**Age groups, years**		
31–35	13	37.14
36–40	3	8.57
41–45	7	20
46–50	5	14.28
51–55	4	11.42
56–60	2	5.71
≥60	1	2.85
**Years of clinical experience**		
1–5	8	22.85
6–10	15	42.85
11–15	9	25.71
16–20	1	2.85
≥20	2	5.71
**Previous training in genetics and/or cancer risk prediction tools**		
Yes	1	2.85
No	34	97.14
**Specialisation or special interest**		
Women’s health	7	20
Breast cancer	4	11.42
Genetics	1	2.85
Women’s health and breast cancer	4	11.42
Women’s health, breast cancer, and genetics	4	11.42
None of the above	15	42.85
**GP Practices**		
**Region**		
West Midlands	5	14.28
Yorkshire and the Humber	5	14.28
South East	5	14.28
London	4	11.42
South West	4	11.42
North West	4	11.42
East of England	3	8.57
North East	3	8.57
East Midlands	1	2.85
Wales	1	2.85
**Category**		
Urban	25	71.42
Mixed	9	25.71
Rural	1	2.85
**Size (number of patients registered)**		
0–5000	5	14.28
5001–10000	8	22.85
10001–15000	11	31.42
15001–20000	5	14.28
≥20000	6	17.14

1. After completing an online consent form, participants received an email with instructions and the link to access the training on Moodle. To start the training, participants were asked to complete the pre-training evaluation questionnaire on Qualtrics using an embedded link. Completion of the questionnaire was designed to take no longer than 10 minutes and on completion, participants were automatically redirected to Moodle and enabled to start watching the training videos.
2. To complete the training, participants were required to watch six training videos in order (40 minutes in total). On completion, the platform allowed participants to access optional education resources and requested the completion of the post-training evaluation questionnaire on Qualtrics using an embedded link.
3. Completing the post-training evaluation questionnaire was designed to take no longer than 10 minutes.

All activities were completed unsupervised. Training completion was checked using the ‘activity completion report’ from Moodle and data from the questionnaires were received electronically as participants submitted their response via Qualtrics.

### Sample size

The primary outcome was objective knowledge. We estimated that a sample of at least 34 participants would enable us to detect a 2-point increase in objective knowledge on scale of 0–10, given an estimated pre-training mean of 6 and standard deviation of 4, with an estimated correlation of 0.5, with 80% power and an alpha of 0.05. Changes of a similar magnitude in knowledge about genetics in healthcare contexts have been found in previous training evaluation studies with no significant differences between GPs and practice nurses.^35–37^

### Analyses

After collating and cleaning all quantitative data, we transformed the scores for reversed items included in the 5-option Likert-type responses. There were no missing data. Descriptive statistics were used to summarise data, and mean changes pre- and post- training completion were analysed with a two-sided paired sample *t*-test. All quantitative analyses and visualisations were supported by Microsoft Excel (version 2402).

Qualitative feedback was analysed using content analysis^38^ on Microsoft Word (version 2402). All participants provided answers to both questions. The lead researcher completed the initial analysis and developed the coding categories. A second researcher coded 34% of the data using the existing categories and validated the process.

## Results

### Development of the training

Based on a scoping review of training needs of healthcare professionals in primary care (Supplementary Table S1), we developed an initial content plan and structure for the training. We shared this plan with the CanRisk general practice expert advisory panel and asked for their written feedback on the following: relevance of the contents covered; to confirm whether anything was missing; and to review the flow of the training. Feedback was mostly positive and helped us prioritise contents and refine the structure. Details of the training are shown in Table 1 and the four educational videos are available on www.canrisk.org.

### Evaluation study

#### Participant demographics

Between May and July 2024, 35 participants were recruited. Sixty-three per cent identified as female and 37% as male. Participants were aged between 31 years and >60 years and had between 1 year and >20 years of clinical experience. Only one participant had previous training in genetics and/or cancer risk prediction tools and 57% of participants reported a specialisation or special interest in topics related to the training (that is, women’s health, genetics, breast cancer). The participants’ practices were spread across 10 regions in the UK, were mostly urban and mostly medium size (10 001–15 000 patients) (Table 2).

#### Quantitative

##### Objective knowledge, subjective knowledge, and confidence

**Figure 1.**
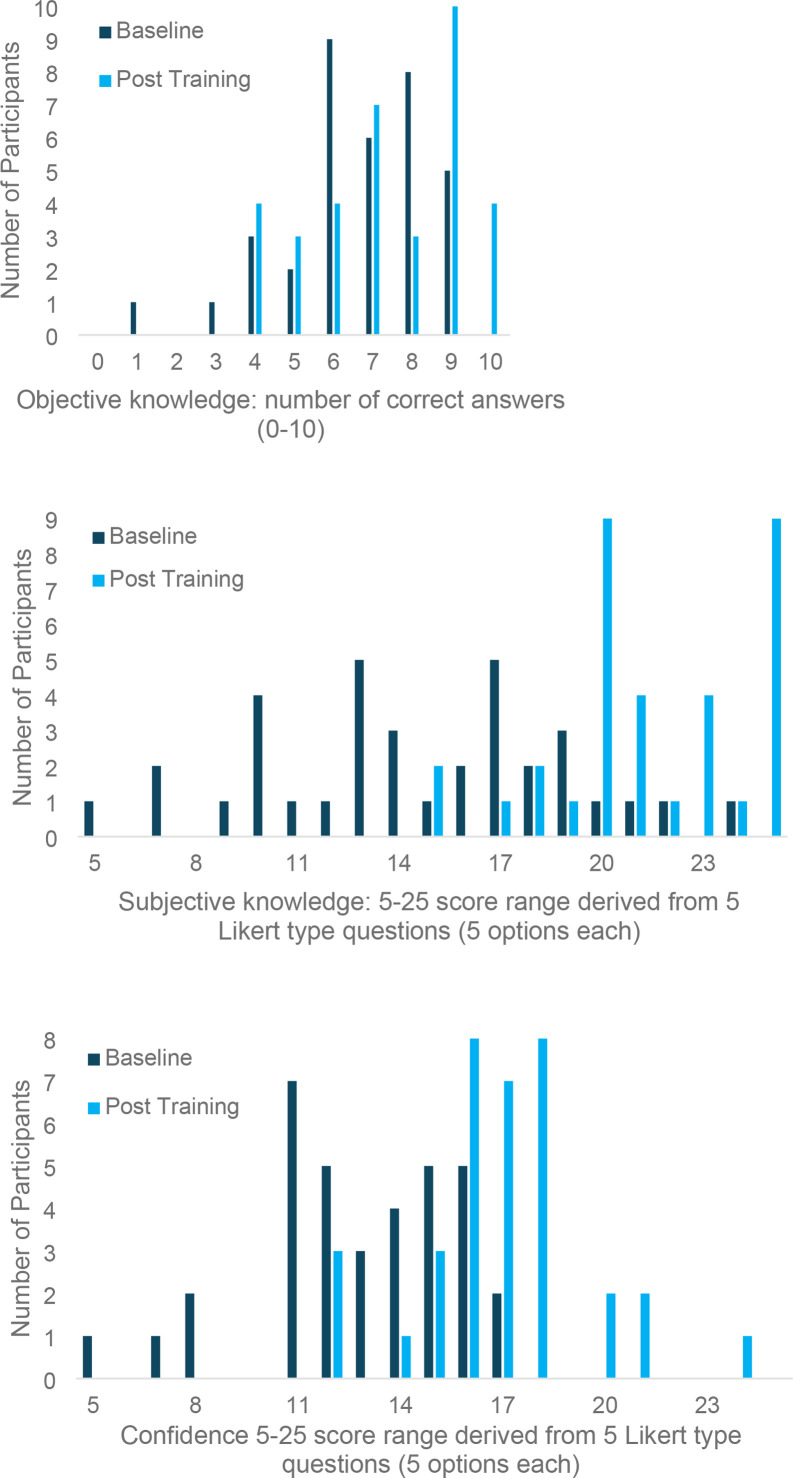
(**A-C**) Pre–post training scores (*n* = 35)

There was a significant increase in objective knowledge, subjective knowledge, and confidence after completion of the online training. Mean objective knowledge increased by 0.77 to 7.37 (0–10 scale, standard deviation [SD] = 1.7, 95% confidence interval [CI] = 0.187 to 1.355, *P* = 0.011) (Figure 1A), with 66% of participants improving their score in the post-training evaluation relative to baseline, 8% showing no change, and 26% showing a decrease in their score. Mean subjective knowledge increased by 6.82 to 21.4 post-training (5–25 scale, SD = 4.9, 95% CI = 5.150 to 8.506, *P*<0.001) (Figure 1B). Mean confidence increased by 4.08 to 16.91 (5–25 scale, SD = 3.8, 95% CI = 2.764 to 5.406, *P*<0.001) (Figure 1C) (Table 3).

**Table 3.** Changes in objective knowledge, subjective knowledge, and confidence pre- and post-training (*n* = 35)

			Paired differences				*t*-test	Significance
					95% confidence interval of the difference			
	Mean pre-training	Mean post-training	Difference in the mean	Standard deviation	Lower	Upper		Two-sided *P*
Objective knowledge	6.600	7.371	0.771	1.699	0.187	1.355	2.686	0.011
Subjective knowledge	14.571	21.400	6.828	4.883	5.150	8.506	8.272	<0.001
Confidence	12.828	16.914	4.085	3.845	2.764	5.406	6.286	<0.001

##### Reaction to the training

**Figure 2.**
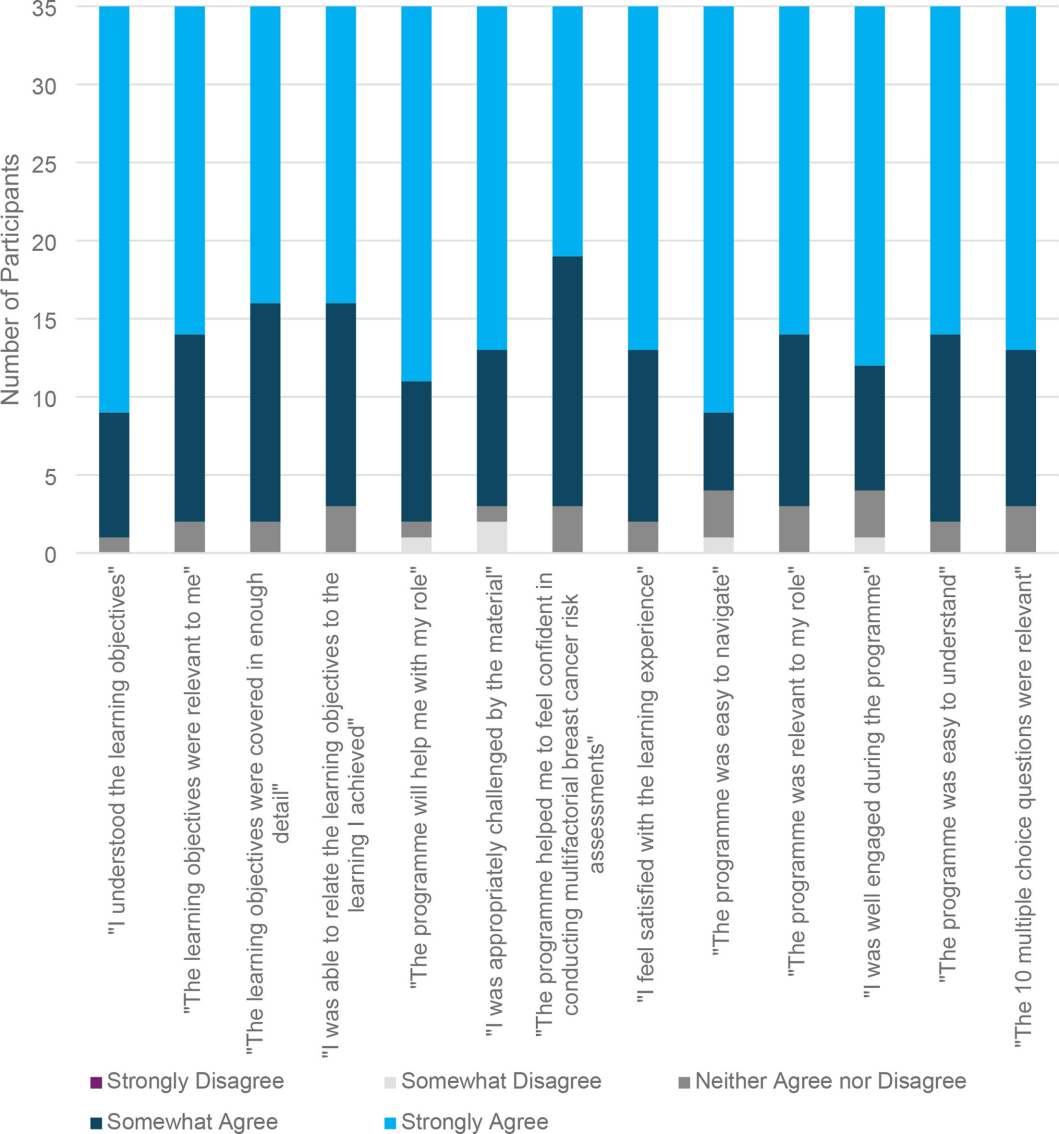
Reactions to the training (*n* = 35)

Quantitative results on satisfaction, engagement, and relevance of the training were very positive with a mean combined score of 58.94 (13–65 scale derived from 13 questions, five-option Likert-type responses each, SD = 6.65) (Figure 2). Overall, 60% of the participants thought the training was ‘Excellent’, 34.3% thought it was ‘Good’, and 5.7% thought it was ‘Average’. No one reported thinking the training was ‘Poor’ or ‘Terrible’, or ‘Didn’t know’.

### Qualitative

When asked about their thoughts on the training, participants highlighted both positive features and opportunities for improvement. These were organised around the following three main categories: the content of the training*;* the presentation of the training*;* and relevance of the training (Table 4). Positive feedback on the three main categories described the training as highly informative and useful, concise, accessible, and clearly structured, as well as relevant to participants’ roles and primary care more broadly. Opportunities for improvement included suggestions to include a demonstration of the CanRisk tool and simulations and case scenarios and providing handouts. The feedback also helped us identify and address some accessibility issues and more general concerns on the part of healthcare professionals, such as time taken to complete a CanRisk assessment and the integration of the tool with clinical record systems.

**Table 4.** Qualitative data analysis summary

Category	Positive feedback	Positive feedback example quotations	Opportunities for improvement	Opportunities for improvement example quotations
The content of the training	Highly informative and useful.	*‘It is an eye-opener programme and very educative.’* (p29 Female PN, North East) *‘Very informative and detailed. Relevant and actionable steps that can be taken by primary care physicians.*’ (p1 Male GP, London) *‘Exceptionally educative and informative.’* (p15 Male PN, South East)	Demonstration of the CanRisk tool.	*‘It was good to understand how to interpret a CanRisk example report, but it would have been useful to also learn a bit more on how to access CanRisk, and a brief section on how to fill in the CanRisk questions in practice.’* (p19 Female GP, East Midlands) *‘The opportunity to complete the CanRisk tool would be good.’* (p34, Female PN, Yorkshire and the Humber) *‘Perhaps a demo of the CanRisk assessment procedure showing each drop-down section.’* (p23 Female PN, South West)
The presentation of the training	Concise and accessible. Clearly structured.	*‘Great succinct online learning, short and concise presentations, simple explanations of risk models and use of CanRisk.’* (p7 Male GP, North West) *‘The training was simple and short but yet contained every detail needed for an improved practice as relating to my knowledge of breast cancer.’* (p21 Female PN, North West) *‘Topic was very well explained in concise and timely manner and objective of learning were clear from start.’* (p9 Male GP, West Midlands) *‘Very helpful focused work, with good section breakdown and rationale for each area of discussion.’* (p17 Male GP, North West)	Including simulations and case scenarios. Providing handouts. Accessibility issues.	*‘More interactive sessions and adding more real-life clinical cases to presentations.’* (p9 Male GP, West Midlands) *‘I think some offline resources to go alongside would be helpful (like "handouts").’* (p18 Female GP, East of England) *‘Some of the accents were more difficult to understand. At times it felt a little rushed with some of the more detailed slides moving on quickly.’* (p34, Female PN, Yorkshire and the Humber) *‘It was difficult to read the slides on a mobile phone.’* (p6 Female GP, East of England)
Relevance of the training	Relevant for primary care healthcare professionals. Applicable to primary care. Completing the training as a good opportunity.	*‘I was not previously aware of the CanRisk tool, and I am glad I am able to use this straightaway.’* (p2 Female GP, West Midlands) *‘It was interesting and appropriate to my role as general practitioner.’* (p4 Female GP, West Midlands) *‘Very happy I had the chance to do this, learnt a lot and feel well informed.’* (p16 Female PN, South West)	Concern about time. Integration with clinical record system.	*‘My main concern is my ability to complete this depth of conversation within a 10-minute appointment slot. I wonder whether there is any scope to develop a questionnaire (or AccuRx type template) to gather the information needed to calculate a patient’s risk so that they can then be bought in for an appointment at a later stage.’* (p26, Female GP, South East) *‘Does it incorporate into GP software such as EMIS and SystmOne? or is there coding available to document it?’* (p2 Female GP, West Midlands)

## Discussion

### Summary

With the recent change to the NICE guidelines providing greater opportunity for multifactorial breast cancer risk assessment to be completed within primary care, it is essential that PCPs are both confident and competent in the use of risk prediction tools. The results provided here suggest that our ‘Multifactorial breast cancer risk assessment in primary care’ online training significantly increases PCPs’ objective knowledge (*P* = 0.011), subjective knowledge (*P*<0.001), and confidence (*P*<0.001) to conduct multifactorial breast cancer risk assessments, and was well received by PCPs.

### Strengths and limitations

To our knowledge, this is the first study reporting the development and effectiveness of a bespoke training programme aimed at increasing the knowledge and confidence of PCPs around multifactorial breast cancer risk assessment in the UK. The development of the training was based on relevant literature (Supplementary Table S1) and the input of PCPs and experts in risk prediction. To improve the generalisability of the evaluation findings, we purposefully recruited a varied sample taking into account participants’ interests, previous training, and specialisation. Still, more than half of the participants reported previous interest or specialisation in a relevant field, more than 65% were between 31 and 45 years of age, and most participants practised in urban areas. A larger and more varied sample may help increase the generalisability of the results. Our study design used two digital platforms (Moodle and Qualtrics); hosting the training and evaluation in one platform would have expedited data collection and limited opportunities for administrative errors. Consistent with other national training programmes, our study focused on levels 1 and 2 of the Kirkpatrick model. As CanRisk and other multifactorial cancer risk prediction tools become more widely used in primary care, future research focusing on real-world long-term scenarios required to assess levels 3 and 4 of the Kirkpatrick model will become available.^29^

### Comparison with existing literature

Our results show that the online training improved PCPs’ objective knowledge, but it especially improved subjective knowledge and confidence. This adds to previous research showing how training can be particularly helpful in improving clinicians’ confidence in genetics, shared decision making, and risk communication.^22,29^ Compared with previous research,^35^ the size of the increase in objective knowledge was smaller, but the baseline mean score was higher and the SD was smaller. The size of increase in subjective knowledge and confidence in our study were higher or comparable with those in previous research.^35^ Our results on ’reaction to the training’ confirm previous research on the effectiveness, acceptability, and practicality of online training methods on topics relevant to cancer risk assessment.^30,31^ More specifically, results on the relevance of the training for primary care and participants’ valuing the opportunity to complete the training are consistent with previous research identifying PCPs’ training needs on basic genetics,^14,15^ genetic testing,^12^ and counselling and risk communication.^23^ Qualitative findings on participants’ concerns about time and the tool’s integration with clinical records system have been reported elsewhere,^39^ and work is underway within the CanRisk programme to address these challenges.

### Implications for research and practice

The results of this study provide evidence to support using the ‘Multifactorial breast cancer risk assessment in primary care’ online training in the context of a study aiming to evaluate the feasibility, acceptability, and psychological impact of providing multifactorial breast cancer risk assessment in primary care.^40^ Based on the results of the evaluation, we have updated the training to include handouts as offline resources and a note specifying that completing the training on a laptop may improve user experience and resolve the main accessibility issue identified. Considering the training needs covered by this e-learning and how well received it was, there is potential for wider implementation. Suggestions from participants on ways to further improve the training (that is, including a demonstration of CanRisk and simulations and/or case scenarios, slowing down the pace of content delivery) will inform potential future versions of the training.

In conclusion, the ‘Multifactorial breast cancer risk assessment in primary care’ online training significantly increased PCPs’ knowledge and confidence to conduct multifactorial breast cancer risk assessments and was well received by PCPs. The support offered by this training will be key to help PCPs consolidate the knowledge, confidence, and motivation to conduct and facilitate shared decision making around a complex, but potentially highly beneficial risk assessment in primary care.

## Data

The dataset relied on in this article is available from the corresponding author on reasonable request.
